# Medical Opinion and Movement

**Published:** 1909-03-20

**Authors:** 


### Medical Opinion and Movement.

The transplantation of tissues and the establishment of the circulation by union of blood-vessels have recently occupied the attention of several investigators in America. Separate contributions by C. E. Guthrie, of St. Louis, and A. Carrel, of New York, appear in the Journal of the American Association on the subject. Both these authors have succeeded in uniting together arteries and veins in animals by means of careful suturing, so as to transmit a free circulation of blood. They emphasise the importance of attention to the minutest details in order to obtain satisfactory results. After occluding the exposed vessels with properly guarded forceps, the ends are carefully pared with sharp scissors and coated inside and out with paraffin oil to prevent drying. A component strand of Chinese twist silk and No. 16 cambric needles are used for suturing, and the threads are kept in paraffin oil. The ends of the vessels are brought together by two or more sutures and then further united by a continuous suture encircling the point of union. Vessels of less than 2 mm. are more difficult to handle than larger vessels. Both Carrel and Guthrie have succeeded in transplanting limbs in dogs, and Guthrie has transplanted dogs' heads with preservation of cerebral and bulbar function. Carrel has transplanted kidneys and spleens with re-establishment of their circulation by vascular anastomosis, and the kidneys have functioned normally. These results are highly interesting in themselves, but it is impossible at present to form any definite conclusions as to what practical value such work may possess in its possible application to the human subject. According to Carrel, it is not absolutely necessary that the transplanted vessel be from the same species of animal, but the best results are obtained when the species are most closely related.

---

Dr. B. Aschner describes in the *Wiener Klinische Wochenschrift* an ocular reflex which has apparently hitherto escaped observation. In the case of a patient under deep anæsthesia who could not be aroused by the usual means, the author had recourse to a practice he had seen performed by Professor Wagner of Jauregg, upon the insane in a state of stupor, consisting in compression of the eyes. Immediately the face of the patient flushed, and at the same time there was a deep inspiration followed by an effort to vomit, and finally the patient opened his eyes. While exerting pressure on the eyeballs, Dr. Aschner examined the radial pulse and was surprised to find that the pulsations tended to disappear. Coupled with the attempts to vomit this diminution of the pulse suggested an irritation of the vagus, either of a reflex nature or possibly by excessive pressure of cerebrospinal fluid. Dr. Aschner then carried out a series of experiments on animals to further elucidate the problem. Pressure on the eyeballs producing the same effect on the pulse with the cranial cavity freely open negatived the cerebrospinal fluid hypothesis. On the other hand the phenomenon was abolished after section of the trigeminal nerve, but remained the same after section of any other of the cranial nerves. It appears, therefore, that the phenomenon is reflex, the trigeminal nerve forming the centripetal path and the vagus the centrifugal. In rabbits this reflex affects chiefly respiration, whereas in dogs and in men it shows itself chiefly by a change in cardiac activity, the pulse disappearing as soon as pressure is exerted sufficiently strongly upon the eye, to reappear again as soon as the pressure ceases. The author states that in anæsthesia the reflex persists longer than the corneal and pupillary reflexes and is easier to obtain in the condition of anæsthesia than waking.

---

The treatment of tuberculous glands of the neck by means of injections is in no way a novel method. Injections of iodoform emulsion in glycerin have been advocated, and successful results have attended their use in the hands of certain operators, but in the majority of cases preference is given to surgical interference and extirpation with the knife. It must be admitted, however, that these operations are often of the most serious and tedious nature, involving a complete dissection of the side of neck and even in the most skilful hands may not be wholly successful. Recurrences, unsightly scars, and cheloids are among the not infrequent consequences. In view of these facts, Dr. Henri Judet's account in *Le Progrès Médical* of his treatment of these conditions by injections of camphorated naphthol is not without interest. In the case of suppurating and caseating glands he considers these injections are especially the method of choice, and even in indurated glands of long-standing, if really tuberculous in nature, he claims considerable success for the treatment. He points out, however, that this last-named form of adenitis is not infrequently secondary to enlarged tonsils and adenoids, removal of which may be followed by complete disappearance of the cervical adenitis. The treatment is somewhat tedious and prolonged, as several injections are required, often as many as 20, at intervals of one to three days. The drugs should be used quite fresh and not more than 0.5 c.c. injected at a time. Larger doses may be followed by signs of syncope. The advantages claimed for the treatment are that complete cure of the condition is obtained without the dangers or fears of operation, and without leaving any scar or trace of the malady. The treatment itself, if carefully carried out, is free from dangers or complications of any sort.

---

Some years ago Dr. Lancereaux advocated the administration of tincture of cantharides for cases of tubular nephritis which do not yield to the ordinary treatment with diuretics. The use of the drug in these conditions was, however, warmly contested on the ground that it excites inflammation of the kidney, and cannot, therefore, cure an already inflamed organ. In a contribution to the *Journal de Médecine* Dr. Lancereaux again returns to the subject and reports a number of cases in which he has obtained remarkable success by the exhibition of this drug in small doses. One case was that of a little girl, aged 8, who developed a severe nephritis after scarlet fever. There were albuminuria, hæmaturia, and cylindrical casts, with vomiting, pallor, œdema of the face and lower limbs, and insomnia, and the total amount of urine excreted in 24 hours varied between 150 and 250 cubic centimetres. The symptoms persisted in spite of treatment for nearly a month, and then one minim of tincture of cantharides was administered. On the following day the amount of urine excreted rose to 600 c.c. For the four following days two minims were given each day, and the amount of urine each day rose successively to 1,000, 1,400, 1,600, and 2,000 c.c. The drug was then stopped, and the patient made rapid progress, and at the end of six weeks left the hospital entirely cured. Similar results followed the treatment in some cases of nephritis in young adults, brought on by exposure to cold and excessive exertion. In each case the symptoms were most severe, especially in regard to the diminished quantity of urine excreted. In these cases, of course, larger doses were given, commencing with 4 or 5 minims and increasing to 12 minims a day for a few successive days. On each occasion the administration of the drug was immediately followed by a rapid increase in the amount of urine excreted and a steady amelioration of all the symptoms to complete cure. These facts, supported by so high an authority as Dr. Lancereaux, will undoubtedly lead to a more extended trial of the drug in such cases.

---

From Texas comes a report by Dr. M. B. Saunders of a drug which has been found extremely valuable in the treatment of amœbic dysentery. This is prepared as a fluid extract or an infusion from the bark of the stem of a plant which grows in Texas and Northern Mexico, and is known locally as goat bush, or chaparro: the scientific name is *chaparro amargoso*, or *castela nicholsoni*. Dr. Saunders prefers the fluid extract in drachm doses every three or four hours, but he has seen cases in which this preparation failed to benefit, whereas the infusion proved immediately successful. The latter form is also that in which the natives themselves employ the drug, imbibing it freely three times a day before meals. The treatment requires to be kept up for some considerable time, but it is said to be extremely successful, and it is even believed that small hepatic abscesses may become sterile under it, though it is admitted that large ones require surgical treatment. It appears there is some difficulty in obtaining pharmacologically active specimens of the drug; and a resinous principle which has been isolated and named amargosin has not yet been fully investigated. The professor of pathology at the University of Pennsylvania, Dr. A. J. Smith, who was formerly of the University of Texas, employs preferably the infusion; and he goes so far as to express the opinion that a case of dysentery which does not react quickly to the treatment is not one of the typical amœbic form.

Mr. W. Taylor publishes in the *Dublin Journal of Medical Science* the details of some cases of gastric disease diagnosed by exploratory laparotomy as malignant, but proved by their subsequent course not to be so. As a matter of experience, this sequence is not so rare as would be supposed, for many of the cases remain unpublished. A curious point is that in each case hydrochloric acid was absent from the stomach. The first patient, aged 48, had for ten months symptoms suggestive of pyloric obstruction of a malignant kind. A movable tumour was felt in the right epigastrium, and this, when exposed by operation, appeared like a typical carcinoma. There were two enlarged glands in the lesser omentum; and as the patient was deemed too feeble for partial gastrectomy, a gastro-enterostomy alone was performed. Three weeks later the abdomen was reopened in order to deal with the tumour, but the latter was found to be soft instead of hard, and to have shrunk from the size of a duck's-egg to that of a horse-chestnut. Nothing, therefore, was done, and five months afterwards the patient was in perfect health and had gained three and a half stone in weight. Another case was similar in essentials; and in a third a growth believed to be malignant was excised, but turned out to be an inflammatory mass surrounding a chronic ulcer. The author protests against the performance of gastro-enterostomy indiscriminately for disorders of the stomach; comments on the small import of a lack of hydrochloric acid in the test meal; and closes with some remarks on the great difficulty in the differential diagnosis of chronic ulcer with tumefaction, and of gastric cancer. In the former case he concludes that gastro-enterostomy alone is not, as a rule, sufficient. He would excise always the ulcer as well.

---

Another paper on gastric diagnosis, with especial reference to cancer is contributed to the *Medical Chronicle* by Mr. H. Upcott. In considering the relative value of various tests, such as that for hydrochloric acid, that for occult blood in the stomach contents and the fæces, the examination of films for micro-organisms, and the enumeration of leucocytes, he concludes that "the presence or absence of free hydrochloric acid in the stomach contents one hour after a test meal is to be regarded as the most important single 'test.'" Still, in his own cases, but three out of six which gave the test turned out malignant; the other three comprised two simple ulcers and a case of ileo-cæcal tuberculosis. If in the fasting stomach there is found no hydrochloric acid, but many saccharomyces and sarcinæ, this is held to be against carcinoma; if a great profusion of bacteria, to be in favour of it. Occult blood in the fæces only may come from a duodenal ulcer: in both vomitus and fæces it is more likely to be from the stomach, and the lesion may be innocent or malignant. Other tests are also discussed, but the summing-up contains the very just observation that a positive diagnosis of carcinoma of the stomach, based upon the described tests, is to be made with much less assurance than a negative one of its absence.

An extremely rare—probably unique—case was recently shown at the New York Post-Graduate School by Dr. Thompson Sweeny, and its occurrence adds one more to the long list of conditions which might lead to confusion in the differential diagnosis of venereal sore. The patient was a woman of 27, married, who presented herself complaining of a single lesion on the inner mucous surface of the right labium minus. The general health was good, and no other patch existed anywhere on the body. The lesion simulated a venereal sore, but, on microscopical examination of scrapings taken from it, the mycelium and spores of the tinea tricophyton were found, and the diagnosis of ringworm became inevitable. It is generally supposed that mucous surfaces are exempt from the attentions of this fungus, but it is evident that this is not invariably the case. The diagnosis must of necessity be of extreme difficulty; indeed, impossible without microscopical examination. The hair follicles in the neighbourhood of the vulva were not attacked, as the patch of infection was entirely confined to the inner surface of the labium.

---

Dr. E. Mansel Sympson, in a letter to the *British Medical Journal*, speaks most favourably of the use of peroxide of hydrogen in the treatment of chilblains. The remedy was apparently recommended first by Courtin, of Bordeaux, and it is, of course, for external application, so that it can be used simultaneously with the administration of calcium chloride or of calcium lactate internally. We have already referred to the usefulness of these calcium salts in doses of about ten grains thrice daily, in curing many chilblains, even when no local applications are used at the same time. Hydrogen peroxide used externally will cure even without internal medication, but in severe cases combined treatment by calcium salts internally and hydrogen peroxide externally would seem to be ideal. Dr. Mansel Sympson says: "My plan is for the patient to bathe the affected parts in peroxide of hydrogen (10 vol. strength) diluted with equal parts of previously boiled water, still hot, for fifteen or twenty minutes, twice daily. This treatment has the additional advantage of being capable of being carried out even if the chilblains are cracked and ulcerated, though it is well to diminish the strength of the peroxide if much pain and irritation is produced by the application. A continuation of this treatment for two or three days in most cases will effect a cure."

---

Dr. E. A. Rothmann, writing to the *Lancet* from Kharkoff, draws attention to a form of treatment which, if results similar to his are obtained by other observers also, will be welcomed by those who are called upon to relieve or cure facial erythema, or "red nose." It consists in the prolonged internal administration of adrenalin. We fear that the results are too good to be true, for we have always supposed that adrenalin becomes entirely decomposed in the stomach; nevertheless the method is not a difficult one to pursue, and it seems well worth trying, not only for "red nose" but also for acne rosacea. Dr. Rothmann's letter is as follows: "The fact that adrenalin is regarded as one of the strongest vaso-constrictors induced me to try its usefulness by internal administration in cases of erythematous tâches of the face ("red nose"). I have made observations on three cases; two of these, demonstrated to the Kharkoff Dermatological Society in 1907, were students of the University, aged 24 and 27 years, and the third was a young lady aged 23 years. In all these cases the adrenalin was given internally in doses of 5 minims of solution of adrenalin chloride 1 in 1,000 in a teaspoonful of water three times daily, half an hour before food. The remedy was continued during five to six months, with short intervals of one or two weeks, and had a remarkable effect on the erythema, which almost entirely disappeared. Up to now, a year and a half after treatment, the redness has not re-appeared, and no bad effect on the heart or blood pressure has been observed."

---

Discussion still rages in the United States as to the correct treatment of ectopic gestation in the various well-recognised stages which it assumes clinically. The conservative principles advocated especially by Robb, Stillwagen, and Simpson in America (and by some surgeons in this country also) have been already summarised in these columns (The Hospital, October 31, 1908, p. 118) in connection with the discussion last year at the American Gynæcological Society. Since the publication of the papers read at that symposium the medical press across the Atlantic has been full of contributions to the subject, many of them by surgeons with very extensive practical experience. The most recent is by Dr. Frank, who considers the whole question of ectopic gestation in a most interesting paper in the American *Journal of Obstetrics*. Dr. Frank is opposed to all expectant treatment for the cases presenting severe or urgent symptoms, the 5 per cent. whom the conservative school prefer to leave until the general condition has improved. He does not agree to the proposition that death never follows hæmorrhage from an ectopic gestation sac, but is in favour of immediate exploration and ligature. As for the remaining 95 per cent. of patients, in whom the diagnosis is established or reasonably suspected, but without symptoms of urgency, he recommends close observation, in a hospital or nursing home preferably, and laparotomy in two or three days if definite improvement has not been noticed. Taking the symptoms themselves, he finds amenorrhœa in exactly 50 per cent.: in the other moiety the menstrual history is of no aid in diagnosis. Spotting, however, occurs in 72.5 per cent., sometimes alternating with profuse metrorrhagia. Pain is almost universal, usually described as "cramp-like" or "cutting" in character. Moderate elevation of temperature is very common, and the pulse-rate is nearly always raised, but often partly through emotional disturbance. A history of fainting is obtainable in a third of the cases, and is a valuable aid in diagnosis.

